# Spatial adiabatic passage of massive quantum particles in an optical Lieb lattice

**DOI:** 10.1038/s41467-019-14165-3

**Published:** 2020-01-17

**Authors:** Shintaro Taie, Tomohiro Ichinose, Hideki Ozawa, Yoshiro Takahashi

**Affiliations:** Department of Physics, Graduate School of Science, Kyoto University, Kyoto, 606-8502 Japan

**Keywords:** Ultracold gases, Quantum mechanics

## Abstract

Quantum interference lies at the heart of quantum mechanics. By utilizing destructive interference, it is possible to transfer a physical object between two states without populating an intermediate state which is necessary to connect the initial and final states. A famous application is a technique of stimulated Raman adiabatic passage, where atomic internal states can be transfered with high efficiency regardless of lossy intermediate states. One interesting situation is a case where the initial and final states are spatially well separated. Quantum mechanics allows a particle to move without practical possibility of being found at the intermediate area. Here we demonstrate this spatial adiabatic passage with ultracold atoms in an optical lattice. Key to this is the existence of dark eigenstates forming a flat energy band, with effective transfer between two sublattices being observed. This work sheds light on a study of coherent control of trapped cold atoms.

## Abstract (web summary)

In spatial adiabatic passage, matter wave is transported between states without populating the intermediate quantum state. Here the authors demonstrate the spatial adiabatic passage using ultracold ^171^Yb atoms in Lieb-type optical lattices.

## Introduction

Interference of probability amplitudes is one of the most significant properties of quantum mechanics. In the seminal work of an electron double-slit experiment^1^, building up of the interference pattern of electron wave function beautifully demonstrated the nature of wave-particle duality. Quantum interference has been intensively utilized especially in the field of precision measurement such as superconducting magnetometer^2^ and atom interferometry^3^, and also lies at the heart of quantum information science.

**Fig. 1:**
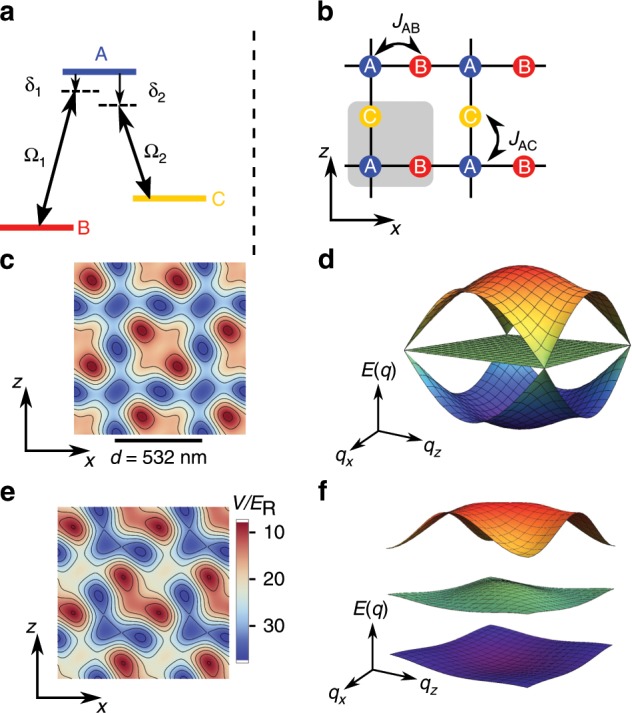
Illustration of the physical system. **a** A *Λ*-type three level system. Rabi frequencies *Ω*_1_, *Ω*_2_, and detunings *δ*_1_, *δ*_2_ are shown. **b** A Lieb lattce. The gray shaded region indicates one of unit cells. **c** Potential landscape of an optical Lieb lattice. **d** Energy band structure of the Lieb lattice with no distortion, calculated in the tight-binding limit. **e** A distroted Lieb lattice potential. Shown is the case of *ψ* = (1∕2 + 0.11)*π* and *s* = [(8, 8), (8, 8), 14], which is used in Fig. 2a. **f** Energy bands of the Lieb lattice with distortion. If the phase of the diagonal lattice is shifted, band gaps become large and the Dirac cone disappears.

A three-level system is a minimal example in which quantum interference takes place. Most commonly it is considered in a context of laser coupled atomic levels, and the Hamiltonian for a *Λ*-type system (Fig. 1a) in a rotating frame is written in the form

1 $$H\ =\ \left(\begin{array}{lll}0&{\Omega }_{1}&{\Omega }_{2}\\ {\Omega }_{1}&{\delta }_{1}&0\\ {\Omega }_{2}&0&{\delta }_{2}\end{array}\right),$$

where *Ω*_1_ (*Ω*_2_) denotes a laser-induced Rabi frequency which couples basis states ∣*A*〉 with ∣*B*〉 (∣*A*〉 with ∣*C*〉), and *δ*_1_ (*δ*_2_) is the detuning of the corresponding laser 1 (2). A dark state ${\rm{cos}}\theta | B\rangle -{\rm{sin}}\theta | C\rangle$ (${\rm{tan}}\theta \ =\ {\Omega }_{1}/{\Omega }_{2}$) arises as one of the eigenstates of the Hamiltonian given by Eq. (1) if the Raman resonant condition *δ*_1_ = *δ*_2_ is satisfied. By applying two laser pulses in so-called counter-intuitive order so that *θ* changes from 0 to *π*/2, the dark states smoothly evolve from ∣*B*〉 into ∣*C*〉. This process is well-known as stimulated Raman adiabatic passage (STIRAP)^4–6^, and has been an important technique for robust population transfer between two atomic/molecular states.

Natural interests arise for a special case that the states of interest represent a matter wave of a quantum particle and the initial and the final states are spatially well isolated. Such processes, named spatial adiabatic passage (SAP), offer paradoxical transport without transit^7,8^ where matter waves are transported without populating the intermediate spatial region. Since the concept of SAP was introduced in the context of quantum dots^9,10^ and cold atoms^11^, it has continuously attracted theoretical interests and various possibilities of its application have been discussed^12^. Generalized to two-dimensions, the SAP technique also enables preparation of states with angular momentum^13,14^. It is also interesting to consider the case of interacting many-body systems such as Bose–Einstein condensates^7,15,16^.

Highly controllable and flexible systems of cold atoms are suitable to realize SAP and the above-mentioned applications. Especially, optical lattices provide many sophisticated methods in manipulating cold atoms and both adiabatic and nonadiabatic technique have been developed. The use of multiple sublattices enables us to prepare a variety of excited states, which can be applied to, for example, detect sublattice populations^17,18^ and even create Bose condensate in higher lattice orbitals^19,20^. Note that classical waves traveling through coupled waveguides are known to obey coupled mode equations^21^ which are mathematically equivalent to Eq. (1) and SAP correspondence was demonstrated^22^. However, the original concept, SAP with matter waves of a quantum particle has not been realized.

In the following, we report on the successful realization of SAP by adiabatic control of matter-wave tunneling in an optical lattice. A key for the realization of SAP is the existence of dark eigenstates. Interestingly, dark states also arise in eigenstates for a particle moving in a special kind of lattice structures. Our lattice consists of three sublattices (*A*, *B*, and *C* shown in Fig. 1b), forming a Lieb lattice. It is convenient to take plane waves on each sublattice ∣**q**, *A*〉, ∣**q**, *B*〉, and ∣**q**, *C*〉 with a momentum **q** as a basis set. The existence of nearest-neighbor tunneling *J*_*AB*_ and *J*_*AC*_ induces coupling among these basis states given by ${{\mathcal{T}}}_{AB}({\bf{q}})\ =\ -2{J}_{AB}{\rm{cos}}({q}_{x}d/2)$ and ${{\mathcal{T}}}_{AC}\ =\ -2{J}_{AC}{\rm{cos}}({q}_{z}d/2)$. The resulting Hamiltonian can be written as a 3 × 3 matrix form

2 $$H\ =\ \sum _{{\bf{q}}}{H}_{{\bf{q}}},\ {H}_{{\bf{q}}}\ =\ \left(\begin{array}{lll}{E}_{A}&{{\mathcal{T}}}_{AB}({\bf{q}})&{{\mathcal{T}}}_{AC}({\bf{q}})\\ {{\mathcal{T}}}_{AB}({\bf{q}})&{E}_{B}&0\\ {{\mathcal{T}}}_{AC}({\bf{q}})&0&{E}_{C}\end{array}\right).$$

Now the analogy to a *Λ*-type system (1) is obvious: momentum-dependent couplings play a role of Rabi couplings in a three-level system and detunings can be mimicked by energy offsets *E*_*A*_, *E*_*B*_, and *E*_*C*_ of each sublattice. All these parameters can be controlled by changing the lattice depth along each direction, which enables us to realize a coherent scheme to transport atoms among these sublattices. Our SAP technique with optical lattice provides an effective way to adiabatically prepare atoms in higher orbitals, which is potentially applicable to a certain class of lattice structures with a dark energy band.

## Results

### Expermental realization

Our experimental setup is similar to that described in refs. ^23,24^. In brief, an optical Lieb lattice is (Fig. 1c) created by the combination of a 2D staggered superlattice with *d*/2 = 266 nm spacing and a diagonal lattice with $\sqrt{2}\times 266$ nm periodicity. The resulting potential *V*(**x**) is given by $V({\bf{x}})/{E}_{R}\ =\ -{s}_{{\rm{short}}}^{(x)}$ ${{\rm{cos}}}^{2}(2\pi x/d)-{s}_{{\rm{short}}}^{(z)}$ ${{\rm{cos}}}^{2}(2\pi z/d)-{s}_{{\rm{long}}}^{(x)}$ ${{\rm{cos}}}^{2}(\pi x/d)-{s}_{{\rm{long}}}^{(z)}$ ${{\rm{cos}}}^{2}(\pi z/d)-{s}_{{\rm{diag}}}$ ${{\rm{cos}}}^{2}[2\pi (x-z)/d+\psi ]$, where *E*_*R*_ = *ℏ*^2^∕2*m*(*π*∕*d*)^2^ is the recoil energy for the long lattice. Below we specify a lattice potential by *s*-parameters $s\ =\ [({s}_{{\rm{short}}}^{(x)},{s}_{{\rm{short}}}^{(z)}),({s}_{{\rm{long}}}^{(x)},{s}_{{\rm{long}}}^{(z)}),{s}_{{\rm{diag}}}]$ and the relative phase of the diagonal lattice *ψ*. Basically, tunneling amplitudes are determined by the depths of short lattices (${s}_{{\rm{short}}}^{(x)}$, ${s}_{{\rm{short}}}^{(z)}$) and the other lattice depths are responsible for the energy offsets *E*_*A*_, *E*_*B*_, and *E*_*C*_. In our experiment, we use fermionic ^171^Yb with a small scattering length  −0.14 nm to avoid interaction effects. The use of fermions introduces a complexity arising from the finite momentum spread due to the Pauli principle. In the absence of interactions and harmonic confinement, the dynamics conserves quasimomentum and states initially having well-defined quasimomentum **q** evolve within a subspace spanned by three plane waves ∣**q**, *A*〉, ∣**q**, *B*〉, and ∣**q**, *C*〉.

Adiabaticity of a process associated with a certain momentum is governed by the band gaps among the corresponding eigenstates. This implies that, for a Lieb lattice, adiabaticity cannot be maintained around the corner of the Brillouin zone where a Dirac cone exists (Fig. 1d). To overcome this problem, we slightly deform the lattice structure by shifting the phase from an isotropic condition *ψ* = *π*/2. The effectiveness of this scheme can be understood from the potential landscape shown in Fig. 1e. The deformation reduces the inter-unit-cell tunneling, therefore each cell becomes more like an isolated triple well. As a result, the momentum dependence of the dispersion curves is reduced and the Dirac cone is eliminated as shown in Fig. 1f. Mathematically, this modifies the coupling term as ${{\mathcal{T}}}_{AC}\to {e}^{i{q}_{z}d/2}({J}_{AC}+\delta J)+{e}^{-i{q}_{z}d/2}({J}_{AC}-\delta J)$ (similar change applies for ${{\mathcal{T}}}_{AB}$), where *δJ* denotes the imbalance between inter- and intra-unit-cell tunneling. For *δJ* = 0, ${{\mathcal{T}}}_{AC}$ along the Brillouin zone boundary (*q*_*z*_*d* = *π*) vanishes throughout the process. Introduction of *δJ* ≠ 0 can also suppress the breakdown of transport along this line.

### Spatial adiabatic passage

**Fig. 2:**
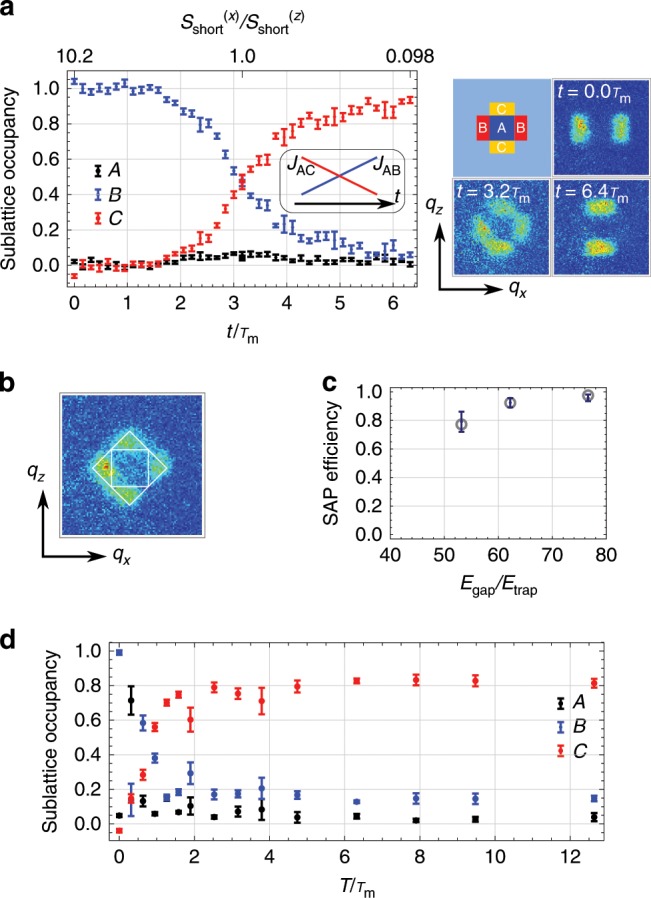
Realization of spatial adiabatic passage. **a** Time evolution of sublattice occupancies *N*_*A*_, *N*_*B*_, and *N*_*C*_ during a SAP process. Time is rescaled by *τ*_*m*_ = 0.63 ms. Samples of absorption images of Yb atoms taken in the experiment are also shown in the right hand side, corresponding the distribution at the initial (*t* = 0.0*τ*_*m*_), half (*t* = 3.2*τ*_*m*_), and final stage (*t* = 6.4*τ*_*m*_) of the process. Inset shows the temporal change of the coupling parameters. Error bars represent standard deviation. **b** Band-mapping image at the half point of the SAP process (**a**). Boundaries for the first and second Brillouin zone are indicated by white lines. **c** Dependence of SAP efficiency on the ratio *E*_gap_/*E*_trap_. The gray circles represent the theoretical estimation with trap. Error bars represent standard deviation. **d** Adiabaticity of the SAP process. Sublattice occupancies after SAP are shown as a function of total sweep time *T*. Slightly low SAP efficiency compared to that in (**a**) is attributed to the instability of the lattice phase *ψ*. Error bars represent standard deviation.

A matter-wave analogue of a STIRAP in the Lieb lattice is to transport atoms between two sublattices (*B* and *C*) by a counter-intuitive temporal change of tunneling amplitudes. Throughout this process, the intermediate sublattice *A* is not populated because the state adiabatically follows a dark state ${\rm{cos}}{\theta }_{{\bf{q}}}| {\bf{q}},B\rangle -{\rm{sin}}{\theta }_{{\bf{q}}}| {\bf{q}},C\rangle$, with ${\rm{tan}}{\theta }_{{\bf{q}}}\ =\ {{\mathcal{T}}}_{AB}({\bf{q}})/{{\mathcal{T}}}_{AC}({\bf{q}})$. First we load a sample of 1.2 × 10^4^ atoms at a temperature as low as 0.3 of the Fermi temperature into the optical Lieb lattice. In the loading stage, the potential on a *B*-sublattice is made much deeper than those of the others (*s* = [(27.7, 0), (0, 16), 14]) to ensure that the initial state is only populated by *B*. After that, we quickly change the lattice depths to [(38.9, 3.8), (8, 8), 14] in 10 μs. This is a starting point of SAP process, where the tunneling *J*_*AB*_ is much smaller than *J*_*AC*_. To achieve a high tunneling rate, overall lattice depths are set relatively shallow, leading to an unwanted direct tunneling *J*_*BC*_. We suppress *J*_*BC*_ by increasing the diagonal lattice depth beyond the equal-offset condition, i.e., *E*_*A*_ > *E*_*B*_ = *E*_*C*_. As long as the two-photon resonance condintion *E*_*B*_ = *E*_*C*_ is maintained, the dark state persists and SAP can be accomplished. We adiabatically sweep the lattice depths toward another limiting configuration [(3.8, 38.9), (8, 8), 14], passing through the intermediate point [(8, 8), (8, 8), 14] corresponding to the potential shown in Fig. 1c. The time evolution during this process is monitored by mapping sublattice populations onto band populations followed by a standard band mapping technique^23,24^. The obtained time-of-flight images suffer from the blurring of the Brillouin zone boundaries due to unavoidable nonaddiabaticity of the band mapping procedure and a harmonic confinement of the system. For a quantitative analysis of sublattice populations, we take a set of basis images in which all atoms reside on a specific sublattice and determine sublattice occupancies by projecting images onto each basis. Figure 2a shows the time evolution of sublattice occupancies *N*_*A*_, *N*_*B*_, and *N*_*C*_ during the SAP process. The time scale is renormalized by *τ*_*m*_ = 2*πℏ*/*E*_gap_ where *E*_gap_ is the energy gap averaged over entire Brillouin zone at the half point of the process. Important features specific to SAP are well reproduced: initial population on the *B*-sublattice, *N*_*B*_, is smoothly transfered into *N*_*C*_, but *N*_*A*_ does not show increase throughout the process. From the final population we evaluate the efficiency of the process to be *E* = [*N*_*C*_(*T*) − *N*_*C*_(0)]/*N*_*B*_(0) = 0.95(2), where the specified uncertainty is statisical. Note that the estimated SAP efficiency includes an uncertainty of  ~ 10%, originated from the detection method (see Methods). During the SAP process the state is certainly following the dark state as one can see in the direct band-mapping image shown in Fig. 2b. Here, the quasimomentum distribution in the lattice is revealed instead of the sublattice distributions shown in Fig. 2a. The concentration of the atomic distribution inside the second Brillouin zone indicates the state is kept in the second, flat energy band which consists of the dark states. Usually, occupation of a certain energy band is accomplished by filling up all lower bands. The above SAP process provides an efficient way to prepare a nonequilibrium many-body state in which all fermions reside on the flat band of the Lieb lattice and the other bands are empty.

In our system, the SAP efficiency is limited by the existence of a trap-induced harmonic confinement. This causes inhomogeneity of energy offsets, especially around the edge of an atomic cloud. We change the ratio of *E*_gap_ to *E*_trap_ = *mω*^2^*d*^2^/2 with *ω* denoting the mean trap frequency on *x*–*z* plane, and monitor the change of SAP efficiency. As seen in Fig. 2c, the trap reduces the SAP efficiency. The behavior is well reproduced by the theoretical calculations, in which we simulate the process with an artificial detuning of 0.2*E*_*R*_ equivalent to the typical potential gradient caused by the trap.

We examine adiabaticity of the SAP process by changing a total sweep time *T* (Fig. 2d). As naively expected, for *T*∕*τ*_*m*_ ≲ 1, adiabaticity is broken and significant populations in *A* and *B* are observed. Once the adiabatic condition is fulfilled for large *T*∕*τ*_*m*_, the final state is kept almost constant, with a large population in *C* regardless of *T*. This behavior is characteristic to a robust adiabatic process, in contrast to Rabi oscillations driven by a direct tunneling coupling.

### Bright state transport

**Fig. 3:**
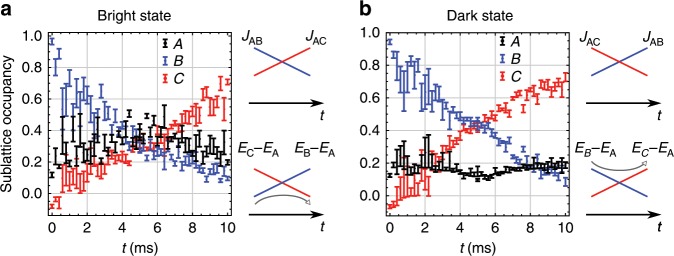
Bright vs. dark state transport. **a** Transport from the *B*- to *C*-sublattice through the bright state. A schematic of the temporal change of the coupling constants is also shown in the right hand side. **b** Transport through the dark state. After loading to the *B*-sulattice, we exactly reverse the potential sweep applied in **a**. Error bars represent standard deviation.

In atomic three-level systems on the one hand, an intermediate ∣*A*〉 state generally suffers from significant loss due to spontaneous emission. One of the advantages for STIRAP is that the loss through ∣*A*〉 state can be avoided by keeping the dark state. On the other hand, SAP with cold atoms does not suffer from any losses through intermediate states, which enables us to perform efficient transport through bright states where ∣*A*〉 is significantly populated. We design an intuitive potential sweep for this bright state transport for the optical Lieb lattice. The sweep [(25, 30), (7.6, 8.4), 14] → [(8, 8), (8, 8), 14]  → [(30, 25), (8.4, 7.6), 14] involves not only ${s}_{{\rm{short}}}^{(x,z)}$ (tunneling) but also ${s}_{{\rm{long}}}^{(x,z)}$ (detuning) to improve the transport efficiency. Figure 3a shows the resulting time evolution during this sweep. Due to the requirement of minimizing unwanted excitations at the beginning of the sweep, initial localization on *B* is not perfect in this experiment. However, the nature of the bright state transport is clearly visible as the increase of *N*_*A*_ around the half point. As a reference, we also perform a counter-intuitive scheme under the same condition. In Fig. 3b we start with a sample localized on *B*, but apply the potential sweep in a time-reversed way compared with that of Fig. 3a to transport atoms through the dark state. This scheme is essentially equivalent to the SAP process demonstrated in the previous section, except that the state does not always remain dark because *E*_*B*_ − *E*_*C*_ changes during sweep. Similar performance of transport efficiency is obtained, but the behavior around the half point of the process is qualitatively different. At this point, the state becomes exactly dark and the *N*_*A*_ certainly shows its minimum, in contrast to the bright state transport. The feasibility of accessing both bright and dark state transfer manifests the flexibility of our system in quantum state engineering.

### Autler–Townes doublet

Electromagnetically induced transparency (EIT)^25^ is also an important process in a three-level system. In an EIT experiment, there is strong optical coupling between *A* and *C* which causes splitting of the *B* → *A* transition by the Rabi frequency, known as Autler–Townes doublet^26^. As a result, the state *B* becomes transparent for laser light driving the *B* → *A* transition at a frequency region between the doublet.

**Fig. 4:**
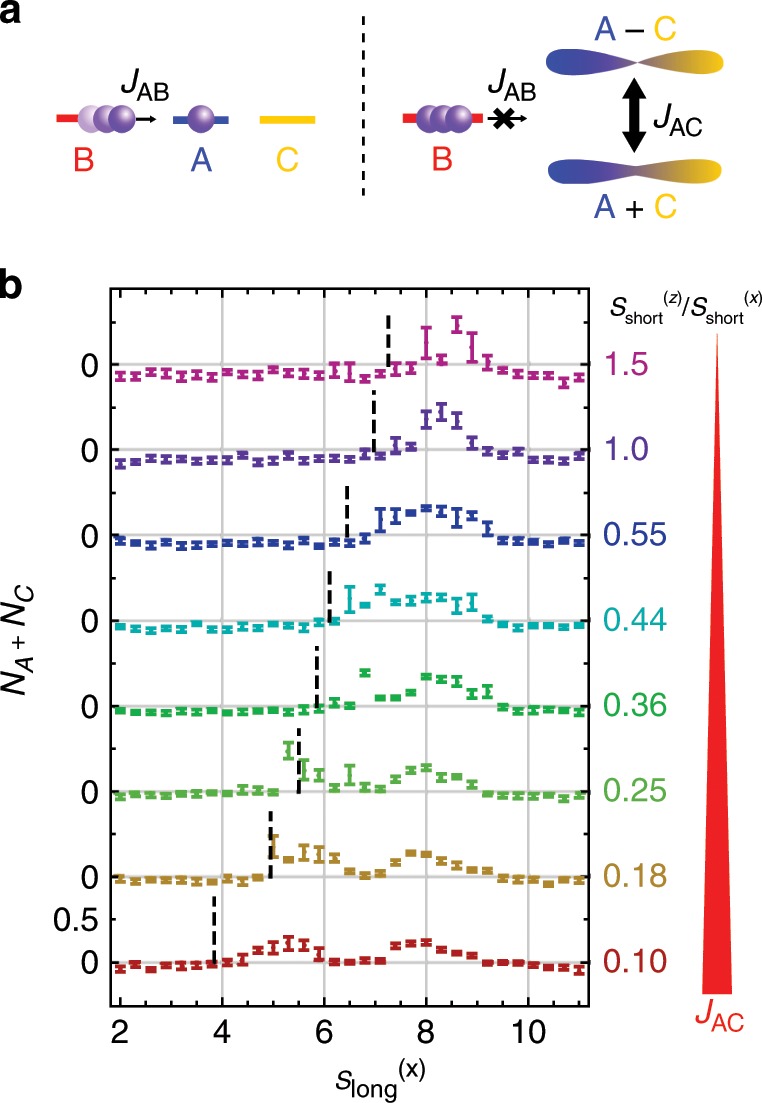
Analogue EIT experiment. **a** Schematic of the experiment. (left) In the absence of *J*_*AC*_, the weak tunneling coupling *J*_*AB*_ results in a fraction of atoms in *A*. (right) In the presence of strong *J*_*AC*_, tunneling only occurs when the energy resonates to bonding *A* + *C* or antibonding *A* − *C* orbitals, split by coupling *J*_*AC*_. **b** Tunneling spectrum after a hold time of 1.8 ms, at the lattice depths of $[(40,{s}_{{\rm{short}}}^{(z)}),({s}_{{\rm{long}}}^{(x)},8),9.5]$ with *ψ* = (1∕2 + 0.07)*π*. Fraction of atoms which tunnel from the *B*-sublattice is shown. Vertical dashed lines show the rough estimation of the points where *E*_*A*_ = *E*_*B*_ is satisfied, based on a harmonic approximation of the lattice potential. Error bars represent standard deviation.

To investigate a matter-wave analogue of EIT physics, we carry out a measurement similar to a pump–probe experiment, as depicted in Fig. 4a. As before, we first prepare an initial state localized on *B*. Then we allow weak tunneling coupling *J*_*AB*_ (~0.1*E*_*R*_) and after a fixed time, a fraction of atoms that tunneled into *A* or *C* is measured. Figure 4b shows a set of tunneling spectra. In each spectrum we scan the detuning ${s}_{{\rm{long}}}^{(x)}$ which determines the energy difference *E*_*B*_ − *E*_*A*_ (=*E*_*B*_ − *E*_*C*_). As we increase the coupling *J*_*AC*_ (decrease ${s}_{{\rm{short}}}^{(z)}$), the spectrum drastically changes: for negligible *J*_*AC*_, we can observe a single peak corresponding to *B* → *A*, whereas a clear doublet structure appears for *J*_*AC*_ ≫ *J*_*AB*_. The double peaks originate from tunnelings to *A* + *C* and *A* − *C* orbitals which are separated by the amplitude of tunneling coupling *J*_*AC*_. The overall shift of the spectrum is caused by the change of the short lattice depth ${s}_{{\rm{short}}}^{(z)}$. While the short lattice creates the same potential curve for all sublattices, its effect on *E*_*A*_, *E*_*B*_, and *E*_*C*_ slightly differs depending on the configuration of other lattices. We estimate the shift of zero-point energy by a harmonic approximation. Whereas it fails to predict the position of resonance center, the tendency of the shift is well captured. In this experiment, the system can be regarded as weakly coupled two lattices: B and AC. The former is almost dispersionless and the latter has strong dispersion if *J*_*AC*_ is large. For the data with the largest splitting, the band width of the AC lattice amounts to 1.5*E*_*R*_, which is comparable to the observed linewidth. In principle, the lineshape has information of the density of states in the AC lattice. However, in the current setup, the detailed structure has been smoothened out by inhomogeneity of the trap (<0.2*E*_*R*_). In addition, the technical instability of the relative phase *ψ* also affects the spectrum: it causes the fluctuations of the peak center and results in the large error bars in the data with small *J*_*AC*_.

In a typical EIT spectrum, a sharp dip can be observed even when the doublet splitting is smaller than the natural linewidth. This implies occurrence of coherent population trapping (CPT)^27^ of a dark eigenstate. In the case of our system with no loss mechanism, CPT does not occur and hence a sharp EIT dip does not appear. However, the observed behavior exactly corresponds to a pump–probe detection of Autler–Townes doublet which is commonly observed in atom-field systems. As a future direction, it is interesting to introduce site-dependent loss (by e.g., high-resolution laser spectroscopy) and study CPT and related phenomena.

## Discussion

The three-sublattice structure of the Lieb lattice has remarkable analogy to *Λ*-type three level systems in quantum optics. By using this analogy and dynamical controllability of tunneling amplitudes, spatial addiabatic passage between two sublattice eigenstates was performed. We also observed a matter-wave analogue of Autler–Townes doublet in a tunnleing process from a sublattice into a strongly coupled pair of sublattices. The demonstrated techniques are useful to prepare exotic many body states in optical lattices. For example, at the half point of the SAP process in the Lieb lattice, all atoms are located on the flat band. This might be a general scheme applicable to other lattices with flat bands. Involving higher lattice orbitals is also interesting in connection with generation of angular momentum studied in ref. ^14^. In addition, recent advances in fine potential engineering^28^ combined with site-resolved imaging of lattice gases^29^ will greatly enlarge the application of SAP in cold atomic systems.

## Methods

### Sample preparation

A quantum degenerate gas of ^171^Yb is produced by sympathetic evaporative cooling with fermionic ^173^Yb, in a crossed dipole trap operating at 532 nm^30^. After evaporation, remaining ^173^Yb atoms are cleaned up by illuminating resonant laser light on the ^1^*S*_0_ ↔ ^3^*P*_1_(*F* = 7∕2) transition. All the data presented in the main text are taken with spin unpolarized samples.

### Optical Lieb lattice

Our optical Lieb lattice consists of a short square lattice operating at a wavelength of 532 nm, a long square lattice at 1064 nm and a diagonal lattice at 532 nm. The lattice beams for two square lattices are retroreflected by common mirrors. Therefore the relative phases between these lattices are determined by the relative frequencies between the short and long laser beams, which are actively stabilized to realize the staggered lattice configuration. The relative phase *ψ* of the diagonal lattice is stabilized by a Michelson interferometer and the uncertainty is estimated to be 0.02*π* during a typical measurement time^23^.

### Sublattice occupation measurent

To project sublattice occupations onto band occupations, the lattice potential is suddenly changed into *s* = [(20, 20), (8, 8), 0]. Without the diagonal lattice, the *x* and *z* directions are decoupled and atoms in *B* (*C*) sublattice are mapped onto the first excited band of the *x* (*z*) direction. Followed by the band mapping technique, atoms distribute over the (1 + 1) dimensional Brillouin zone with the one-to-one correspondence to sublattice occupancies (*N*_*A*_, *N*_*B*_, and *N*_*C*_).

To analyze data, we fit an empirical model function *F*(*q*_*x*_, *q*_*z*_) = *N*_*A*_*f*_*A*_(*q*_*x*_, *q*_*z*_) + *N*_*B*_*f*_*B*_(*q*_*x*_, *q*_*z*_) + *N*_*C*_*f*_*C*_(*q*_*x*_, *q*_*z*_) + *b* with taking *N*_*A*_, *N*_*B*_, *N*_*C*_ and the background level *b* as free parameters. Here, the basis functions *f*_*A*_, *f*_*B*_, *f*_*C*_ are constructed by averaging more than ten absorption images under the condition that almost all atoms localize on a sole sublattice.

### SAP efficiency

The limitation of above occupation measurement is that the Wannier functions for the detection basis *s* = [(20, 20), (8, 8), 0] do not perfectly coincide with those for the other lattice configurations. Therefore sudden change to the detection basis causes  ~10% of atoms excited to the higher bands, which will not be properly analyzed. In designing the SAP sequence, we optimize the SAP efficiency calculated within the subspace spanned by the detection basis. In the sequence shown in Fig. 2, the efficiecncy of 98% is predicted without trap inhomogeneity. However, this value itself does not have physically important meaning because of the arbitrariness of the detection basis. Precise evaluation of the SAP efficiency and improving it close to 100% will require more sophisticated routine of designing sequences together with better detection method (such as quantum gas microscope).

## Data Availability

The datasets are available from the corresponding author on reasonable request.
